# An Exploration of Analgesia Options for Australian Sheep

**DOI:** 10.3390/ani14070990

**Published:** 2024-03-22

**Authors:** Shari Cohen, Emily Foss, Thierry Beths, Gabrielle C. Musk

**Affiliations:** 1 Animal Welfare Science Centre, University of Melbourne, Parkville, Melbourne, VIC 3010, Australia; 2 Melbourne Veterinary School, University of Melbourne, Parkville, Melbourne, VIC 3010, Australia; emilypagefoss@gmail.com (E.F.); thierry.beths@unimelb.edu.au (T.B.); 3 School of Human Sciences, University of Western Australia, Perth, WA 6009, Australia; gabrielle.musk@uwa.edu.au

**Keywords:** analgesia, sheep, pain, ovine

## Abstract

**Simple Summary:**

Sheep may undergo a variety of painful husbandry and disease processes in their lifetime, which negatively impact their welfare. These procedures can cause considerable pain that may be unalleviated due to a lack of pain relief options across many different settings such as farm, clinical, and biomedical contexts. The choice of pain relief may be restricted due to licensing requirements (e.g., Australian regulations) or lack of known effectiveness. In a biomedical setting, a variety of potential pain relief options have been used but not validated for pain relief or safety (human residues or sheep welfare). A review of the farm, veterinary, and biomedical literature was undertaken to identify important gaps in sheep analgesia, pain management, and potential options for pain relief to promote better sheep welfare across these industries.

**Abstract:**

During their lifetime, sheep undergo many painful husbandry and disease processes. Procedures undertaken on the farm, such as tail docking, castration, and mulesing, all cause considerable pain. In addition, sheep may experience painful diseases and injuries that require treatment by veterinary practitioners, and in biomedical research, sheep may undergo painful experimental procedures or conditions. It is important due to ethics, animal welfare, social licence, and, at times, legal requirements for farmers, veterinary practitioners, and researchers to provide pain relief for animals in their care. While there is a heightened awareness of and a greater interest in animal welfare, there remain few licensed and known analgesia options for sheep within Australia. A literature review was undertaken to identify currently known and potential future options for analgesic agents in sheep in farm and biomedical settings. Non-steroidal anti-inflammatories, opioids, local anaesthetics, α_2_ adrenoreceptor agonists, and NMDA receptor antagonists are some of the more common classes of analgesic drugs referred to in the literature, but few drugs are registered for use in sheep, with even fewer proven to be effective. Only six analgesic product formulations, namely, lignocaine (e.g., Numocaine^®^), Tri-Solfen^®^, ketamine, xylazine, and meloxicam (oral transmucosal and injectable formulations), are currently registered in Australia and known to be efficacious in some types of painful conditions in sheep. The gap in knowledge and availability of analgesia in sheep can pose risks to animal welfare, social licence, and research outcomes. This article presents a summary of analgesic agents that have been used in sheep on farms and in clinical veterinary and biomedical research settings along with details on whether their efficacy was assessed, doses, routes of administration, indication for use, and pain assessment techniques (if any) used. The outcome of this research highlights the challenges, gaps, and opportunities for better analgesia options in sheep.

## 1. Introduction

Sheep in Australian meat and wool production enterprises undergo painful husbandry and disease processes throughout their life. Most lambs are ‘marked’ between 4 and 12 weeks of age [1]. Surgical or painful procedures undertaken at this time may include earmarking, tail docking, castration, and/or mulesing. These procedures cause considerable pain with impacts on animal welfare, especially if performed without any analgesia [1]. Sheep also experience painful conditions such as shearing cuts, mastitis, foot abscesses, dystocia, and flystrike, for which they may or may not be treated by a veterinarian or farmer. The lack of administration of pain relief for painful husbandry practices entrenched within Australia’s sheep farming industry is waning in public acceptability [2]. Phasing out these procedures or at least providing analgesia is a practice more commonly being advocated for by both industry and the public. The Australian Wool Innovation (AWI) industry organisation in 2017 released a Merino Husbandry Practices Survey, which reported that up to 85% of lambs were likely to receive some form of pain relief when mulesed. AWI also reported that up to 42% of producers used pain relief for tail docking and castration [3]. A 2018 Meat and Livestock Australia (MLA) survey found that up to 39% of producers would be willing to use pain relief for marking if it were available and effective [4]. There is an increasing demand from local and global retail brands as well as industry markets for more ethical, higher-welfare-produced wool and meat from producers committed to using pain relief. Markets and retailers typically grant a price premium to more ethical, higher-welfare products, offering producers greater financial benefits with greater market access for their products. According to the Australian Wool Exchange (AWEX), data reveal that wool from sheep treated with pain relief receives a premium that often offsets the cost of any pain relief administered [5,6].

In biomedical research, various procedures including orthopaedic, reproductive, cardiac, and abdominal surgeries are performed on sheep [7,8,9]. The use of pain relief in these procedures can ensure better animal welfare and higher ethical standards, promote the Three Rs, and minimise potential impacts on research outcomes. In addition, researchers, institutions, and animal ethics committees are working under Australian legislative requirements published by the National Health and Medical Research Council Code of Practice for the Care and Use of Animals for Scientific Purposes (2013 and 2018) to consider and manage pain and distress. These codes also require any choice of analgesic regimen to be consistent with current best veterinary or medical practice, appropriate for the species and life stage of the animal, and compatible with the purpose and aims of the project [10]. In Australia, the type and dose of pain relief given to sheep in biomedical trials can include licensed and unlicensed drugs, with the latter often extrapolated from veterinary drug use in other species or from human medicine [11]. The literature provides an array of analgesic agents at various doses administered for various conditions to sheep. However, many of these analgesic agents have not been investigated for safety (for sheep or in meat) or efficacy, and in some of these publications, methods of pain assessment are not disclosed. Even if pain assessment in sheep is performed via sheep-specific and generic parameters [12], this does not ensure that the analgesic choice selected is effective, appropriate, or safe. This issue poses potential animal welfare concerns and risks confounding experimental work due to unmitigated pain or side effects of these therapies [13,14,15,16]. To achieve best practice in pain relief, research, and sheep management, further research is needed to ensure that preventative and multi-modal analgesic regimes are fit for purpose.

There are also additional ethical responsibilities, societal demands, and potential legal requirements of veterinary practitioners, farmers, and researchers to provide adequate pain relief to animals in their care. Heightened public awareness and interest in animal welfare are key drivers to ensure that appropriate pain relief is administered to farm and experimental animals. Increasing societal concern for animal welfare is reflected in the public statement of the Royal Society for the Prevention of Cruelty to Animals (RSPCA) that ‘all future systems must identify and adopt humane husbandry and management practices that do not cause pain, suffering or distress to animals. In the interim, best practice pain relief must be used’ [17]. Specifically for those working in the Australian sheep industry, the Australian Animal Welfare Standards and Guidelines for Sheep state that lambs must have analgesia for many common painful husbandry procedures from 6 months of age onwards [18]. When lambs are under 6 months old, pain relief is not required but still recommended. Additionally, livestock South Australia (an industry body), Victorian state regulations, and Tasmanian state regulations all mandate pain relief for mulesing and recommend it for all other invasive procedures from various ages [19]. The future sustainability of the sheep industry will likely require further investment, development, and formal experimental trials of suitable products for safe administration and effective analgesia.

Non-steroidal anti-inflammatory drugs (NSAIDs), opioids, local anaesthetics, α_2_ adrenoreceptor agonists, and N-*methyl*-D-aspartate (NMDA) receptor antagonists are classes of analgesic drugs reported in the literature. Depending on the national jurisdiction (e.g., the European Union), analgesia options may be different, limited, or unavailable [20]. In Australia, there are only six analgesic formulations registered (also known as ‘licensed’) for use in sheep: lignocaine (2%); Tri-Solfen^®^ (lignocaine hydrochloride 40.6 g/L, bupivacaine hydrochloride 4.2 g/L, adrenaline (as acid tartrate) 24.8 mg/L, and cetrimide 5 g/L); ketamine (as hydrochloride 100 mg/mL); xylazine (as hydrochloride 20 mg/mL); and oral transmucosal and injectable formulations of meloxicam (20 mg/mL) [21]. Their product registration is as follows: lignocaine is a local anaesthetic registered for use since 1998; Tri-Solfen^®^ was registered in 2011 and is a topical anaesthetic and antiseptic solution; xylazine is an α_2_ adrenoreceptor agonist registered since 1998 [22]; ketamine is an NMDA receptor antagonist registered since 1994 [22]; meloxicam, a non-steroidal anti-inflammatory drug (NSAID), has been registered in its injectable form since 2016;the oral transmucosal formulation, known as Buccalgesic^®^ and, more recently, Butec^®^, is the most recent analgesic drug to be registered for sheep, receiving approval in 2017 [22]. The paucity of effective, registered (permitted), and available products for sheep analgesia poses animal welfare concerns and limits best practice across all jurisdictions in the wider sheep industry.

The aim of this review of analgesic agents used in Australian sheep on farms and in veterinary clinics and biomedical research settings is to identify the possible large array of known and potential analgesic drugs. There are potentially far more future analgesic options that could be available or viable to alleviate pain in sheep if further research, appropriate pain assessment, and safe registration are undertaken. The intent of this review is to offer a starting point to highlight these options as well as promote, encourage, and improve sheep analgesia and welfare across biomedical, veterinary, and farming enterprises.

## 2. Materials and Methods

**Figure 1:**
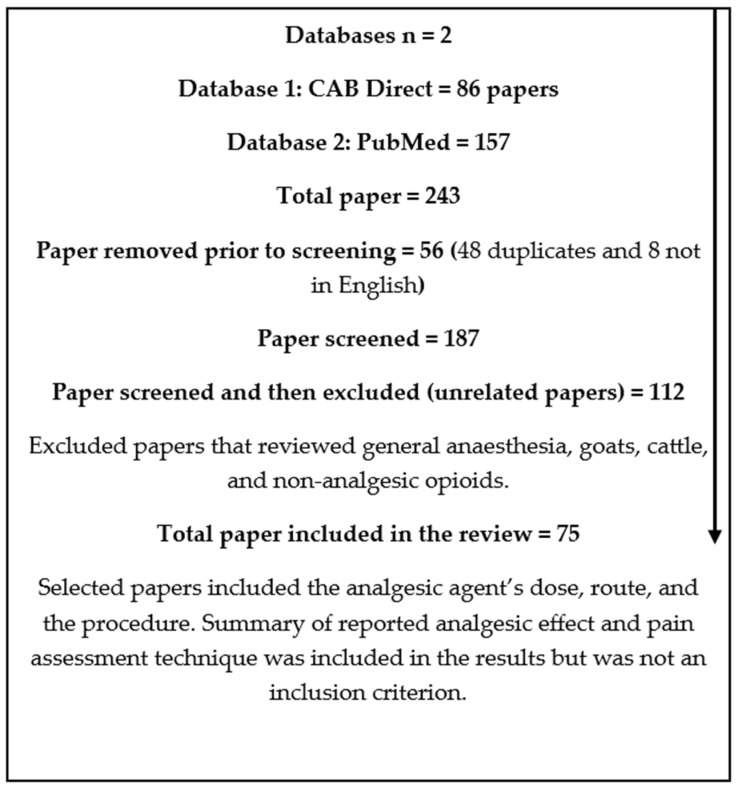
Database review process.

A structured approach to the review was undertaken, as outlined in Figure 1. The electronic literature databases CAB Direct and PubMed were searched from 2010 to March 2022 for the following key terms: analgesia, local anaesthetic, pain relief, opioid, NSAID, ovine, sheep, lamb, ewe, and ram. Further databases were not included in the search due to frequent overlap of articles across databases. Only full-text articles in English or translated into English were included, as the authors’ primary language is English, and non-English articles could not be confirmed to match the information presented in the abstract or used to extract additional information required for review. The criteria for article inclusion required publications to include the analgesic dose, route given, and purpose for analgesic use in sheep either on a farm or in a biomedical research setting. Confirmation and evaluation of pain assessment was not a criterion for inclusion, as the review sought to outline both potential and known options for sheep analgesia rather than assess analgesic effectiveness. The quality and impact factors of journals were not included or used as a criterion for inclusion or exclusion due to the exploratory nature of the review. Two hundred and forty-two (242) articles were found to meet the criteria for inclusion and downloaded into Endnote X9 (Clarivate, Philadelphia, PA, USA). A small selection of hand-picked known information on sheep analgesia methods found using the standard literature review search method were also included.

Papers that were deemed unrelated and therefore excluded were those that focused primarily on general anaesthesia, non-target species (goats or cattle), or non-analgesic opioids. A total of 75 articles met the criteria for review. The results were categorised into five tables by drug class. The analgesic drug classes were NSAIDs, opioids, local anaesthetics, α_2_ adrenoreceptor agonists, and other miscellaneous drugs (e.g., paracetamol and ketamine). Details of drug action, dose, route, indication, summary of analgesic effect, pain assessment method used, and the number of sheep involved in the study were included. The details on drug ‘action’ highlight the pharmacokinetic differences between drugs within their class. The ‘dose’, ‘route’, and ‘summary of analgesic effect’ sections show the variation in these methods of administration between studies. The ‘indication’ for use lists any painful or potentially painful procedures or disease states experienced by sheep. The use of a ‘pain assessment method’ was the assessment tool or constellation of indicators used to identify pain to determine if any pain assessment method was used. The effectiveness of the method used to identify pain was not assessed, as this was outside the scope of the paper. The ‘number of sheep’ was included to show study size.

In several studies, analgesics were administered as part of a surgical anaesthesia protocol and were not the sole focus of the study. These study designs could cause interpretation difficulties, as the primary purpose was not to study analgesic effect. Only information on the reported analgesic agent or regimen was recorded, as the intent of the review was to identify drugs being used for analgesic purposes in sheep.

## 3. Results

The results demonstrated that a far greater number of analgesic drugs and/or regimens (32) have been used for analgesia in sheep than the six currently licensed formulations available in Australia. Multiple studies (21) attempted to utilise multimodal analgesia techniques. Three studies used analgesic drugs for a disease process rather than a procedure.

### 3.1. Non-Steroidal Anti-Inflammatory Drugs (NSAIDs)

Seven NSAIDs were identified in the reviewed literature: ketorolac, meloxicam, flunixin, diclofenac, ketoprofen, carprofen, and phenylbutazone (Table 1). Of these seven, only meloxicam is registered for use in sheep in Australia. Meloxicam was also the most common NSAID used and was utilised in three different multimodal NSAID combinations. The multimodal NSAID combinations were meloxicam with lignocaine, meloxicam with Tri-Solfen^®^, and flunixin with lignocaine. The table below outlines the literature reviewed.

A total of 11/28 studies did not report pain assessment methods. Routes of administration across drugs included the following: ketorolac–intravenous; meloxicam–intravenous, subcutaneous, transmucosal, and intramuscular; flunixin–intravenous, subcutaneous, intramuscular, and oral; diclofenac–topical; ketoprofen–intravenous, intramuscular, and oral; carprofen–oral; and phenylbutazone–oral.

### 3.2. Opioids

Seven opioids were identified: tramadol, buprenorphine, morphine, methadone, fentanyl, remifentanil, and oxycodone (Table 2). No opioid is currently registered for use in sheep in Australia. Fentanyl was the most used opioid and was found in six studies, and three multimodal combinations were reviewed. These were tramadol/lignocaine, buprenorphine/ketamine, and methadone/bupivacaine. The table below outlines the literature reviewed.

A total of seven out of twenty-two studies did not report pain assessment methods. Routes of administration included the following: tramadol–intravenous, intramuscular, transdermal, subcutaneous, and epidural; buprenorphine–intravenous, intramuscular, and subcutaneous (SR-only); morphine–intravenous, intramuscular, and epidural; methadone–intravenous and epidural; fentanyl–intravenous and transdermal; remifentanil–intravenous; and oxycodone–epidural.

### 3.3. Local Anaesthetics

The use of five local anaesthetics were identified: lignocaine, bupivacaine, levobupivacaine, procaine, and ropivacaine (Table 3). Of these, lignocaine is the only local anaesthetic registered for use in sheep in Australia. Lignocaine was also the most studied local anaesthetic, including eight multimodal combinations: lignocaine/xylazine, lignocaine/morphine, lignocaine/adrenalin, lignocaine/tramadol, bupivacaine/morphine, bupivacaine/lignocaine, bupivacaine/methadone, and bupivacaine/fentanyl and Tri-Solfen^®^. The table below outlines the literature reviewed.

A total of two out of twenty-nine studies did not report pain assessment methods. Routes of administration included lignocaine–intra-tissue, epidural, subcutaneous, paravertebral, intramuscular, and nerve blocks; bupivacaine–epidural, paravertebral, and nerve blocks; levobupivacaine–epidural; procaine–intra-tissue and subcutaneous; ropivacaine–epidural and nerve block; and Tri-Solfen^®^–topical.

### 3.4. α_2_ Adrenoreceptor Agonists

Five α_2_ adrenoreceptor agonists were identified: clonidine, xylazine, medetomidine, dexmedetomidine, and detomidine (Table 4). Xylazine is the only α_2_ adrenoreceptor agonist registered for use in sheep in Australia. Medetomidine was the most used α_2_ adrenoreceptor agonist, including two multimodal combinations. The latter were clonidine/lignocaine/buprenorphine, and dexmedetomidine/lignocaine. The table below outlines the literature reviewed.

A total of 2/8 studies did not report pain assessment methods. Routes of administration included the following: clonidine–intrathecal; xylazine–intravenous and intramuscular; medetomidine–intravenous, oral, and intraperitoneal; dexmedetomidine–intravenous and epidural; and detomidine–intravenous.

### 3.5. Other Analgesia

In the recent literature, the use of fourteen analgesic drugs or drug combinations in sheep were identified outside of the drug classes in Table 1, Table 2, Table 3 and Table 4. These were metamizole, ketamine, racemic ketamine, magnesium sulphate, proglumide, diltiazem, nifedipine, verapamil, L-AP_3_, D L-AP_3_, salicylic acid, paracetamol, and amitriptyline (Table 5). Of these fourteen, ketamine was the only drug registered for use in sheep in Australia. There were two multimodal combinations: ketamine/lignocaine and ketamine/magnesium sulphate. The table below outlines the literature reviewed.

A total of four out of seven studies did not report pain assessment methods. Routes of administration included the following: metamizole–intravenous; ketamine/racemic ketamine–subarachnoid and epidural; magnesium sulphate–epidural; proglumide–intracerebroventricular; diltiazem–intracerebroventricular; nifedipine–intracerebroventricular; verapamil–intracerebroventricular; L-AP_3_–intracerebroventricular; D L-AP_3_–intracerebroventricular; salicylic acid–intravenous and oral; paracetamol–intravenous and oral; and amitriptyline–intravenous, epidural, and intrathecal.

## 4. Discussion

### 4.1. NSAIDs

The mechanism of action of NSAIDs is to reduce the synthesis of prostaglandins by inhibiting cyclooxygenase (COX) enzymes in the arachidonic acid pathway [89]. NSAIDs have been shown to have anti-inflammatory, anti-pyretic, and analgesic effects. There was only one drug banned in Australia for use in livestock that was found in this review (phenylbutazone [90]) with the remainder of drugs either registered or potentially able to be used off licence/off label. Meloxicam remains the only NSAID registered for use in Australia for sheep and is available in transmucosal oral and injectable formulations. Both formulations were found in the studies reviewed. The use of 1.0 mg/kg dose of meloxicam was frequently used across all studies except for two studies which used a lower than recommended dose of 0.5 mg/kg [31,32]. An analgesic effect was not recorded when meloxicam was given at this lower dose, and it is unclear whether this lower dose would offer effective pain relief. Therefore, the use of 1.0 mg/kg remains the recommended dose based on the available literature. The timing of the administration of meloxicam varied. However, manufacturer guidelines state pain relief can be effective for up to 24 h. Most studies gave a single dose of meloxicam at the time of the painful procedure. Metacam^®^ also has a broad claim for the alleviation of pain and inflammation which includes any conditions causing inflammation and pain in sheep [21]. It can therefore be prescribed to sheep with painful disease processes such as flystrike, mastitis, foot rot, and shearing cuts in addition to other painful conditions. Three studies recorded the use of an NSAID to alleviate a painful disease process rather than a procedure [27,28,29]. Meloxicam was used for post-partum analgesia although its analgesic effect was not recorded [35]. Flunixin was used for footrot analgesia but was found to have no significant effect on footrot induced lameness [40]. Ketoprofen was also given to reduce pain associated with polyarthritis caused by *Erysipelothrix rhusiopathiae* but its analgesic effect was not recorded [47].

While multimodal analgesia is currently recognised as best practice for lambs undergoing lamb marking in Australia [1], of the three studies that used multimodal analgesia, [26,32,91] only the combinations of Tri-Solfen^®^ with meloxicam and lignocaine and meloxicam are registered for use in sheep (see Table 1). The combination of meloxicam and Tri-Solfen^®^ provided some level of analgesia in most studies [23,24,33]. It should be remembered that Tri-Solfen^®^ is only effective on open wounds. Therefore, it is thought to be suitable for mulesing and knife docking but not suitable for marking (castration and/or tail docking) with rubber rings or similar non-open-wound procedures [92]. As an alternative, the registered meloxicam and lignocaine combination can be used for rubber ring marking methods [34].

The use of drugs confirmed to provide analgesia in some types of painful procedures can be used to manage other painful disease processes on farm under veterinary supervision. Given the paucity of information and inconsistent numbers of formally assessed studies in sheep analgesia, this option may be feasible if there is clear communication with the sheep owner on the use of unlicensed products and a plan for the management of the animal in a farm context where withholding periods must be adhered to. Studies demonstrating NSAIDs are effective at relieving pain associated with naturally occurring diseases are limited, and future research should capitalise on opportunities to demonstrate efficacy. More research to assess the potential frequency, clinical analgesic effect, and refined dosing intervals is required to validate pain relief for both painful procedures and disease processes on farms. Additionally, to ensure withholding periods are appropriate with increased frequency or prolonged dosing regimens. Further research would be required and could be used to approve future prolonged drug dosing regimens across the wider sheep industry to offer more sustained pain relief and improve animal welfare. Drugs that may be of most interest for analgesic use individually or as part of multi-modal analgesia and/or research could include meloxicam, ketoprofen, flunixin, ketorolac, and carprofen.

### 4.2. Opioids

The opioids included in the review were full µ-opioid receptor agonists (morphine, tramadol, methadone, remifentanil, oxycodone), partial µ agonists (buprenorphine), and κ agonists (fentanyl). Opioid receptors are distributed in the periphery, spinal cord, and brain. Opioids combine reversibly with these receptors and alter the transmission and perception of pain. In addition to analgesia, opioids can cause side effects such as sedation, dysphoria, euphoria, and excitement [89]. All studies were for biomedical research procedures. No opioids are currently registered for use in sheep in Australia. Much of the information on opioid analgesia and pain relief validation methods in sheep is extrapolated from other species and human medicine. As evident in Table 2, there remains a large variation in doses, usage, and efficacy between studies. The use of opioids for analgesia in sheep should, therefore, be interpreted and used with care. More studies on the use of opioid dose, and frequency are required to review and confirm of analgesic effectiveness before assuming regimens are clinically suitable for sheep [11].

Fentanyl had the greatest number (6) of publications found in this review. Fentanyl was used intravenously and transdermally in the papers reviewed. Five of the studies using fentanyl patches assessed efficacy of its analgesic effect [48,54,57,61,62]. In the literature reviewed, only fentanyl patches were used transdermally. This finding contrasts with the use of transdermal analgesia, in small animal veterinary clinical practice where fentanyl patches as well as lidocaine and buprenorphine patches can be used for pain relief post-operatively in orthopaedic and laparotomy surgeries [93]. The multimodal combinations of tramadol/lignocaine, buprenorphine/ketamine, and methadone/bupivacaine were all validated for pain relief [52,57,60]. Unfortunately, due to the potential expense and possible risks of human abuse of opioids, it is unlikely opioids will become commonly available for pain relief in farming enterprises. Any potential registration of opioid drugs in sheep would also require the development of appropriate withholding periods to avoid any residues in animals intended for human consumption. However, the use of opioids for the treatment of more invasive and painful procedures is a likely important option in biomedical research. Given these animals do not typically enter the food chain there is minimal risk to human food safety and potentially lower opportunity for misuse as animals are typically held in a highly controlled and regulated environment. If opioids were found to be effective and registered for use in sheep, it would offer the opportunity for uplift and more multi-modal regimens in sheep undergoing painful procedures or conditions. Opioids that may offer the most potential for use or further exploration individually or as part of multi-modal analgesic options could be methadone, fentanyl, morphine, buprenorphine, oxycodone, and remifentanil. Sheep may then be routinely provided with a higher standard of pain relief more akin to small animal and human patients. Procedures such as fracture repair in stud sheep, caesarean sections, or other painful procedures could be performed with better analgesia and contribute to improved animal welfare.

### 4.3. Local Anaesthetics

Local anaesthetics block the transmission of nociceptive impulses in the periphery to the brain [94] to create a local anaesthetic effect in the area of injection and the surrounding tissues innervated by targeted nerves. Nearly all papers listed assessed the local anaesthetic for pain relief and recorded an analgesic effect. Local anaesthetics have been used in both biomedical research and on-farm. Lignocaine is currently the only single-agent local anaesthetic registered for use in sheep in Australia. This differs from other countries’ requirements such as in the European Union where lignocaine is not available (versus procaine) for use production animals [20]. In all three studies that utilised a pre-calibrated 1.5 mL subcutaneous dose of lignocaine via the Numnuts^®^ device, analgesic effects were confirmed when the device was correctly used [64,65,66].

All Tri-Solfen^®^ studies were performed as part of farm studies. There were no studies of Tri-Solfen^®^ use in a biomedical research setting. Interestingly, one study sprayed 1.5 mL Tri-Solfen^®^ directly onto Orf virus lesions [77]. While pain relief was not confirmed in this trial, it could be tested in the future as an option for painful disease states with open wounds. More work should continue to adapt and where possible modify current registered products such as Tri-Solfen^®^ and Numocaine^®^ to promote best practice and maximise the opportunity for analgesia in sheep.

Due to the relatively fast onset of action and short duration of effect, local anaesthetics are often used as part of a multimodal analgesia regime. These types of drugs can also be combined with other more long-lasting analgesics. In the studies reviewed, only the meloxicam/lignocaine and lignocaine/xylazine combinations are registered for use in sheep. There were also several unregistered combinations used for pain relief with potential effectiveness across the literature. Combinations found were lignocaine/morphine, lignocaine, lignocaine/tramadol, bupivacaine/morphine, bupivacaine/lignocaine, bupivacaine/methadone and bupivacaine/fentanyl [11,46,48,50,52,58,66,68,70,72,73,74,76] Meloxicam/lignocaine combinations can be used for various lamb marking procedures (including rubber rings) whereas meloxicam/Tri-Solfen^®^ combinations are only appropriate for open wound procedures such as mulesing and hot-knife tail docking. Lignocaine/xylazine combination is another option that can be administered into the epidural space for caesarean sections or laparotomies for use in veterinary or research procedures. Overall, ropivacaine, lignocaine, procaine, bupivacaine, and levobupivacaine all appear to be potentially viable options for local analgesia. Future trials could assess other combinations of local anaesthetics and/or classes of drugs (e.g., opioids) with meloxicam. The clinical importance of unregistered drug combinations could also be studied further and registered to offer greater options and potential effectiveness for pain relief on farms as well as in research settings.

### 4.4. α_2_ Adrenoreceptor Agonists

α_2_ adrenoreceptor agonists bind to α_2_ adrenoreceptors on vascular smooth muscle, inducing contraction and vasoconstriction [95]. α_2_ adrenoreceptor agonists are commonly used sedative agents in livestock, but have also demonstrated analgesic effects particularly at sub-sedative doses [1]. In veterinary clinical practice, they often form part of a pre-medication anaesthesia protocol due to their combined sedative and analgesic effects. Xylazine is can also be used for epidural anaesthesia in combination with lignocaine [74]. Xylazine is the only α_2_ adrenoreceptor agonist currently registered for use in sheep in Australia. This contrasts with the literature reviewed which identified a range of α_2_ adrenoreceptor agonists (clonidine, xylazine, medetomidine, dexmedetomidine and detomidine) being used in biomedical research settings. Many of these are yet to be formally trialled for effectiveness or administration/regimen optimised. Multiple studies across the literature also noted the common sedative effects of these drugs [80,81]. The majority of studies did not report use of α_2_ adrenoreceptor agonists as a primary agent to treat painful procedures or conditions. However, in some studies it was administered to test analgesic properties via skin and muscle pricks, thermal or mechanical threshold. Appropriate dosing is key with these drugs as risks are associated with α_2_ adrenoreceptor agonists used at higher doses in sheep such as pulmonary oedema and late gestation abortions [96]. Nonetheless, the use of α_2_ adrenoreceptor agonists at smaller doses may prove to be a beneficial adjunct to pain management and/or as premedication for analgesic purposes. Further studies investigating α_2_ adrenoreceptor agonists are required to assess timing and optimal dose for effective potential analgesic effect rather than anaesthetic effects across different dosing regimens.

### 4.5. NMDA Receptor Agonists and Other Drugs

Table 5 summarises drugs that were not classified into any of the previous categories. Ketamine was also reported in the literature in both veterinary and biomedical procedures as a general anaesthetic and analgesic. Both ketamine/lignocaine and ketamine/magnesium sulphate combinations were validated to provide analgesia [44,84]. Similar to most opioids, ketamine’s highly regulated Schedule 8 classification in Australia and profound anaesthetics effects may make it more appropriate on farm for veterinary-only use and/or biomedical research settings [97]. However, unlike other potent analgesics (e.g., opioids) found to be used in this review, it is already registered for use in sheep in Australia. The benefit of this means it has immediate potential to be a used as an adjunct to pain relief at both higher and/or lower doses for painful conditions or when administered as part of an analgesic or anaesthetic regimen for painful procedures. Similar to other drugs found in this review, further studies are still required to evaluate effectiveness, dose rates, timing, frequency, and appropriate routes of administration.

A collection of ‘other drugs’ identified were found within a single biomedical research study investigating the voltage-dependent calcium channel inhibitors of diltiazem, nifedipine, verapamil, proglumide, L-AP_3_ and DL-AP_3_. In this particular study, all the drugs listed were thought to provide visceral analgesia in mechanically induced duodenal distension [85]. Therefore, these drugs may be useful for other types of painful visceral conditions. Salicylic acid, paracetamol, and amitriptyline were also used in other studies but without any analgesic assessment described. Additional research may demonstrate these drugs could be new options or novel applications for pain relief and animal welfare improvement in sheep or possibly other ruminants.

### 4.6. Limitations

This review was undertaken to identify potential analgesic drugs, combinations, regimens, and options used to (potentially) alleviate pain in sheep via the use of scientific databases and grey literature. It is recognised that although many of the drugs utilised may not have been comprehensively investigated or shown to successfully and consistently alleviated pain, the information collated provides a broad list of potential drugs candidates and starting points for drug regimens for future investigations. A key limitation of this study was in the search strategy utilised as it was not feasible to identify all analgesia studies in sheep using the presented search methods. The search strategy was intentionally limited to the use of target words and did not include all known synonyms. While this prevented a higher number of inappropriate or irrelevant results, it may have missed some research-only publications and did miss some of the known textbooks or online formularies which may have listed additional drugs and/or drug regimens [98,99,100].

Additionally, the search criteria omitted publications prior to 2010 and after March 2022, such the more recent use of mint terpenoid L-carvone in sheep [101]. Due to the lack of published studies specific to analgesia in sheep found in the search, and from authors’ knowledge, a small selection of published and grey literature information that fit the criteria for inclusion was also included. It is important to note this study did not fully capture drugs registered in all other countries and did not include the most modern human analgesics developments, such as tapentadol [1]. Some of these drugs might be of value to explore when developing new studies testing analgesics in sheep. Finally, a full review of the analgesic agents and pain assessments strategies utilised in sheep was outside of the scope of this study. Therefore, there remains a wealth of further opportunities available for future publications and research to build upon this review.

### 4.7. General Discussion

The current estimated number of sheep in Australia is 74 million [102]. All of these animals will undergo painful husbandry procedures at some stage in their lifetime. Herein is an enormous opportunity and responsibility for farmers, researchers, animal ethics committees, and veterinarians to improve the welfare of millions of animals through better analgesic practices. Despite the obligation for the provision of analgesia for good animal welfare, only six commercial products (lignocaine (2%), Tri-Solfen^®^ (lignocaine hydrochloride 40.6 g/L, bupivacaine hydrochloride 4.2 g/L, adrenaline (as acid tartrate) 24.8 mg/L and Cetrimide 5 g/L), ketamine (as hydrochloride 100 mg/mL), xylazine (as hydrochloride 20 mg/mL), and oral transmucosal and injectable formulations of meloxicam (20 mg/mL)) are registered to alleviate pain in Australian sheep. Only three multimodal combinations (meloxicam/lignocaine, meloxicam/Tri-Solfen^®^, xylazine/lignocaine) are registered despite the importance of multi-modal analgesia as part of best practices in analgesia for moderately to severely painful procedures [1]. In addition to physically painful conditions or procedures, sheep can also experience painful disease processes. However, only three studies were found that trialled the use of pain relief for a disease process rather than a procedure [40,47,77]. Assessing and validating analgesics for painful procedures and conditions is an essential requirement for good sheep welfare across farm, veterinary clinical and biomedical research settings. There has been minimally publicly available known interest across the meat, livestock, veterinary and biomedical research industries to seek registration of new or novel analgesics products in the last 5 years. The most recent analgesic drug registration for sheep was meloxicam (Buccalgesic^®^ in 2017 [22] and Butec^®^ in 2023 [103]) and there are no other types of drugs known to the authors at this time undergoing testing for registration purposes. Only one topically non-drug analgesic option using cooling via the device CoolSense [87] for mild pain has been studied, but it is yet to be registered for animal use. There is still much more work to explore and required, with many opportunities for collaboration across biomedical research, veterinary clinical and farming industries to bridge the gaps in sheep analgesics.

The aim of this review was to improve the health and welfare of sheep in farming, biomedical and veterinary practices by exploring potential opportunities for analgesics in the scientific literature against the currently approved drugs in Australia. Literature on Australian registered drugs were predominantly found in the context of farming while most of the use of unregistered drugs were found in the biomedical research context. The review demonstrates there is a far larger array of potentially effective analgesics in sheep in comparison to the few available registered products. The use of these non-registered drugs is permitted in many biomedical studies since these animals would not be allowed to and are highly unlikely to exit research facilities prior to humane killing or euthanasia. While this may be the case in Australia and other jurisdictions, this can contrast with other international regulations such those found in the United Kingdom may prevent the use of a more appropriate drugs (cascade system) or in Europe where the use of some analgesics may not be easily permitted even biomedical settings [20]. There is a high likelihood that out of all the non-traditional, unregistered or formally untested drugs described in this research that some may prove to be important alternatives or primary agents in alleviating on-farm, biomedical and veterinary clinical management of pain in sheep. Therefore, there is a need for more research into these and other analgesics to ensure the availability of suitably safe, tested, effective and registered analgesic products to promote better welfare and ensure refinement of research outcomes in sheep.

The information from the sheep biomedical literature shows that there is clearly a potential for improved sheep welfare and an opportunity to alleviate pain to a potentially greater extent and/or beyond the approved drugs in Australia. However, on review of these publications, the dosage and route of administration for many unregistered drugs were quite varied. There were also a reasonable number of publications across the different drugs class categories that did not state the method of pain assessment. This is of considerable concern as confirmation of pain relief post-administration of analgesics is foundational to good veterinary clinical practice. For articles that did state the method of pain assessment, it remains unknown if the methods were appropriate for the procedure and context or if any other indicators were utilised to assist in validations of pain relief. There were also concerns regarding the lack of information listed for the frequency and timing of pain relief administered. According to the ARRIVE guidelines [104] pain relief should be utilised where appropriate and disclosed within publications. Unfortunately, this lack of disclosure has been documented historically in other animal studies [105]. Other issues posing animal welfare risks and concerns include the potential low usage or at a minimum lack of disclosure of appropriate multi-modal pain relief for high impact procedures (e.g., orthopaedics). The appropriate use of multi-modal regimens should be further explored and could have improved from of the pain management regimens.

The extrapolation of analgesics across species for similar conditions can be useful in the absence of other more formal science-based evidence. Nonetheless, it is still vital to support and advocate for well-developed sheep-specific analgesic studies. While it is likely sheep will respond similarly to other small ruminants and mammals, there are well known examples in veterinary medicine where some forms of pain relief can be deadly in other species (e.g., cats and paracetamol [106]) or require significantly higher or lower dosages (e.g., meloxicam in cats [107] versus mice [108]). It is also crucial to ensure appropriate analgesic regimens are explored and suitable options identified for sheep during various life stages (e.g., pregnancy, lambs) and for a variety of painful conditions (e.g., mastitis, bloat, castration).

Given the relatively frequent of use of sheep as large animal models in biomedical studies, it is in the spirit of the three Rs and incumbent on animal ethics committees, researchers, the biomedical industry, and associated veterinarians, to consider if adjustments to experimental design could simultaneously capture, advance, and support better analgesic regimens (ancillary research) in sheep. Simple refinements such as ensuring the use of appropriate pain and animal welfare assessment methods as well as their inclusions in publications would be a great first simple step. These considerations should ideally be a prerequisite for animal ethics committee approval for any sheep undergoing potential painful procedures. Additionally, many of these biomedical studies collect and utilise blood as well as other tissue samples which may be able to be re-used or re-purposed for used in safety and food animal drug testing residue studies to inform withdrawal times for slaughter and safety. While this approach may not be suitable or possible in types of biomedical work with sheep, there are myriad of (lost) opportunities that can be captured to advance the knowledge, welfare, and management of pain management and analgesics in sheep. Without further consideration, advancement and focus on ideal pain regimes for sheep, both the biomedical and farming industries are unnecessarily exposed risks to public support (social licence [109]) as well as possible reduced production [26] and research outcomes [109].

This review highlighted a wide array of unregistered potential drugs and doses that could be useful in sheep. Many of these unregistered and/or minimally studied drugs and doses may have been administered under the assumptions that their mode of action and analgesia would be comparable to humans and other mammalian species. There is still a concern that the dosed, frequencies, and use of these drugs may not be optimal or appropriate. Many of the studies included in this review lacked detailed pain assessment strategies or other key animal welfare indicators to enhance validity. Further probable barriers when using pain relief in food-producing species include the potential for human risk of abuse with more potent analgesics (e.g., companion animals), costs, risk of residues in food and dosing frequencies for appropriate analgesia. There may also be challenges in the practicality, applicability, and appropriateness of when these medications would be suitable for farm, biomedical or veterinary clinical use. These studies should be undertaken to support and encourage the registration of analgesic formulations for sheep including those intended for human consumption. Further research and greater encouragement for collaboration across all sheep industries should be undertaken to improve animal welfare and research outcomes to better meet ethical, societal, and legal obligations.

## 5. Conclusions

Good animal welfare, industry, and veterinary practices dictate that pain relief must be administered to animals experiencing pain., Farmers, veterinarians, and researchers are expected and often required to provide best-practice pain relief to animals undergoing painful procedures and disease processes in their care, highlighting current gaps, challenges, and opportunities for better pain relief in sheep including dose rates, routes of administration, indication of use, and any pain assessment strategies utilised. Both current and possible future analgesia options are outlined with key agents identified for further research either as individual drugs or as part of a multimodal strategy to improve sheep analgesia and welfare. Further research should also focus on the assessment of the safety and efficacy of new drugs or new formulations of old drugs, food safety testing and registration of additional analgesic agents to alleviate pain and improve the welfare of sheep in Australia and worldwide across the farming, biomedical research, and veterinary industries.

## Figures and Tables

**Table 1:** NSAIDs used for analgesia in sheep.

Drug	Action	Dose	Route	Indication	Summary of Analgesic Effect	Pain Assessment Method	Sheep (n)	Reference
Ketorolac	Nonselective COX-1 and COX-2 inhibitor	30 mg/sheep SID for 7 days postoperatively	Intravenous	Open heart surgery.	Not recorded.	None.	10	[7]
Meloxicam *	Selective COX-2 inhibitor	1.0 mg/kg	Oral	Surgical mulesing and hot-knife tail docking.	Slower to provide effective analgesia than Tri-Solfen^®^. Superior analgesia was seen when Tri-Solfen^®^ and Buccalgesic^®^ were used together.	Pain avoidance and postural behaviour, cortisol, haematology, and haptoglobin were used.	24	[23]
		1.0 mg/kg postoperatively	Oral	Laparotomy.	Provided similar analgesia to flunixin. Pain was not eliminated.	Sheep grimace scale, behaviour, blood drug concentration, infrared thermography, pressure mat gait analysis, mechanical nociceptive threshold, and vocalization were used.	12	[8]
		1.0 mg/kg	Oral	Hot-knife tail docking and surgical mulesing.	Analgesia evident at the 2 h observation. Pain was not eliminated. Best analgesia was seen when Tri-Solfen^®^ and Buccalgesic^®^ were used together.	Lamb behaviour was observed.	20	[24]
		1.0 mg/kg	Oral	Hot-iron tail docking and knife castration.	Provided substantial analgesia on the day of marking. Some analgesia evident the following morning.	Time to mother up and behaviours were observed.	30	[25]
		1.0 mg/kg	Oral	Tail docking and ring castration.	Reduced lamb mortality between marking and weaning.	Pain-related behaviour, average daily growth, and feed intake were measured.	78	[26]
		1.0 mg/kg	Intravenous	Forelimb pain.	Provided some pain relief.	Leucocyte count, neutrophil/lymphocyte ratio, haptoglobin, force plate pressure, skin temperature, and daily feed intake were measured.	10	[27]
		1.0 mg/kg	Subcutaneous	Sterile acute inflammation in forelimb.	This dose of meloxicam provided significant analgesic benefits to sheep.	Pain-related variables and inflammation-related variables were measured.	12	[28]
		1.0 mg/kg SID preoperatively and every 48 h postoperatively	Oral	Laser ablation of abscess.	Not recorded.	No.	1	[29]
		1.0 mg/kg SID for 10 days	Oral	No procedure. Trial for meat withdrawal intervals	Provides potential analgesia but not for longer than 24 h.	No.	27	[30]
		0. 5 mg/kg SID	Subcutaneous	No procedure.	Analgesic effect not recorded.	No.	6	[31]
		0.5 mg/kg postoperatively	Intramuscular	Rumen fistulation.	Effect not recorded.	No.	13	[32]
		1.0 mg/kg	Subcutaneous	Mulesing.	Minimal to no analgesia.	Behavioural responses were observed.	20	[33]
		15 min preoperatively, 1.0 mg/kg at mulesing	Subcutaneous	Mulesing.	Minimal to no analgesia.	Behavioural responses were observed.	20	[33]
		1.0 mg/kg preoperatively	Intramuscular	Ring castration and hot iron docking.	Meloxicam had no analgesic effect.	Behavioural indicators of pain were recorded.	15	[34]
		1.0 mg/kg on day 1 and day 4 postpartum	Oral	Post-partum.	Not recorded.	No.	19	[35]
		Not recorded	Subcutaneous around scrotum	Castration.	Provided partial analgesia for ring castration.	Behaviour, plasma haptoglobin, cortisol, rectal temperature, haematology, and behaviour were recorded.	12	[36]
		0.5 mg/kg postoperatively	Intravenous	Elective laparoscopy.	Not recorded.	The UNESP-Botucatu composite scale was used.	48	[37]
Meloxicam * and lignocaine *	Selective COX-2 inhibitor and local anaesthetic	0.5 mg/kg meloxicam + 2 mL 2% lidocaine/sheep	Subcutaneous + intra-testicular injection	Castration.	Minimal analgesia.	Electroencephalography, behavioural observations, and eye temperature were recorded.	8	[38]
		1.0 mg/kg + 1 mL 2% lidocaine/sheep preoperatively	Intramuscular + subcutaneous into scrotal neck, spermatic cords, and tail	Ring castration and hot iron docking.	Some indication that meloxicam improved lignocaine’s analgesic effect but did not fully alleviate pain.	Behavioural indicators of pain were recorded.	15	[34]
		5 mL of 2% lidocaine + 2% meloxicam/sheep	Administered together. Diluted in 5 mL saline, then injected SC into scrotal neck, spermatic cords, and tail	Ring castration and hot iron docking.	Analgesic effects were similar to those of the two drugs when administered separately, but the treatment did not fully alleviate pain.	Behavioural indicators of pain were recorded.	15	[34]
Meloxicam (sustained release)	Selective COX-2 inhibitor	1.5 mg/kg	Subcutaneous	No procedure.	Not measured.	No.	6	[31]
		3 mg/kg	Subcutaneous	No procedure.	Not measured	No.	6	[31]
Meloxicam and Tri-Solfen^®^ *	Selective COX-2 inhibitor + (local anaesthetic + sympathomimetic + antiseptic)	1.0 mg/kg 15 min preoperatively + 8–10 mL/sheep	Subcutaneous + topical (on the mulesed area and tail-docking wound)	Ear marking, castration, tail docking, and mulesing.	No analgesia evident in lambs 1.5 h after the procedures	QBA method.	30	[39]
		1.0 mg/kg 15 min preoperatively + 8–10 mL/sheep	Subcutaneous meloxicam administered 15 min before mulesing and Tri-Solfen^®^ applied after mulesing	Mulesing.	Provided analgesia in the first 6 h post-mulesing.	Behavioural responses were observed.	20	[33]
		1.0 mg/kg + lambs 5–10 kg 6 mL, 11–15 kg 8 mL, 16–20 kg 10 mL, >20 kg 12 mL	Oral + sprayed onto wounds	Hot knife tail docking and surgical mulesing.	Provided analgesia, but pain was not eliminated.	Lamb behaviour was observed.	20	[24]
		1.0 mg/kg + lambs 5–10 kg 6 mL, 11–15 kg 8 mL, 16–20 kg 10 mL, >20 kg 12 mL	Oral + sprayed onto wounds	Hot knife tail docking and surgical mulesing.	Provided analgesia to surgical mulesing.	Behaviour, cortisol, and postures were recorded.	24	[23]
Flunixin	Nonselective COX-1 and COX-2 inhibitor	1.1 mg/kg on day 1	Intramuscular	Foot rot.	NSAID had no significant effect on recovery from lameness.	No.	16	[40]
		2.2 mg/kg postoperatively	Intravenous	Laparotomy.	Provided similar analgesia to meloxicam. Pain was not eliminated.	Sheep grimace scale, behaviour, blood drug concentration, infrared thermography, pressure mat gait analysis, mechanical nociceptive threshold, and vocalization were used.	12	[8]
		1.0 mg/kg every 24 h	Intravenous	Orchiectomy.	Moderate reduction in pain	Pain was assessed.	6	[41]
		4.0 mg/kg	Oral	Turpentine injection was used as a painful stimulus.	Minimal analgesia was seen.	Pain was assessed.	10	[42]
		4.0 mg/kg	Oral in feed	No procedure.	No.	No.	9	[43]
		5.0 mg/kg	Subcutaneous around scrotum	Castration.	Provided partial analgesia.	Behaviour, cortisol, rectal temperature, haematology, and plasma haptoglobin were recorded.	12	[36]
		1.1 mg/kg every 12 h	Intravenous	Retropharyngeal abscess and tracheostomy.	No.	No.	1	[29]
		1.1 mg/kg SID	Intravenous	Post orchiectomy analgesia.	Effect not recorded.	No.	10	[44]
Flunixin and lignocaine (2%)	Nonselective COX-1 and COX-2 inhibitor + local anaesthetic	1.1 mg/kg + 2% lidocaine at 2.5 mL + 5 mL	Intramuscular Subcutaneous (spermatic cords and scrotal neck)	Burdizzo castration.	Analgesic effect for up to 3 days post-castration.	Multiparametric: behaviour, inflammation, ANS, HPA, andoxidative stress.	24	[45]
		1.0 mg/kg 1 h preoperatively, then every 24 h for 2 days postoperatively + 2 mg/kg preoperatively	Intravenous + intrafunicular	Orchiectomy.	Reduced pain and distress preoperatively and postoperatively.	Pain was assessed.	6	[41]
Diclofenac (1%)	Selective COX-1 and COX-2 inhibitor	Placed around tracheostomy site	Topical (gel)	Tracheostomy.	No.	No.	1	[29]
Ketoprofen	Nonselective COX-1 inhibitor	3.0 mg/kg	Intravenous and intramuscular	No procedure.	Not recorded.	No.	6	[46]
		8.0 mg/kg	Oral	Turpentine injection was used as a painful stimulus.	Minimal analgesia.	Pain was assessed.	10	[42]
		3.0 mg/kg for 3 days	Intramuscular	Polyarthritis caused by *Erysipelothrix rhusiopathiae.*	No.	No.	7	[47]
Carprofen	Selective COX-2 inhibitor	8.0 mg/kg	Oral	Turpentine injection was used as a painful stimulus	Achieved putative therapeutic concentrations within 2 h, but little evidence of therapeutic efficacy was seen.	Pain was assessed.	10	[42]
Phenylbutazone	Nonselective COX-1 and COX-2 inhibitor	1.0 g/sheep the day before and the day of the procedure and for 3 days postoperatively	Oral	Stifle surgery.	Effective analgesia.	Behavioural and physiological parameters were recorded.	30	[48]

* = registered for use in Australia.

**Table 2:** Opioids used for analgesia in sheep.

Drug	Action	Dose	Route	Indication	Summary of Effect	Pain Assessment Method	Sheep (n)	Reference
Tramadol	Weak μ agonist + serotonin reuptake inhibitor	4 and 6 mg/kg	Intravenous	Use of a mechanical nociceptive threshold (MNT) device.	Antinociceptive effects were not detected.	Physiological parameters, blood samples, and mechanical nociceptive threshold (MNT) values were recorded.	6	[49]
		1 mg/kg	Lumbosacral epidural	Needle pricks were used as a painful stimulus.	Analgesia lasted for 318.6 ± 5.08 min and began at 14.29 ± 1.24 min.	Pain was assessed in study.	7	[50]
		2 mg/kg	Epidural	Postoperative caesarean section analgesia.	Analgesia up to 8 h.	Adaptation of the UNESP-Botucatu One-Dimensional Scale for Post-Operative Pain Evaluation in Bovine was recorded.	2	[51]
		100.0 mg/sheep	Intravenous	Postoperative analgesia.	Not recorded.	No.	10	[7]
Tramadol (5%) and lignocaine (2%)	Weak μ agonist and serotonin reuptake inhibitor + local anaesthetic	2 mg/kg and 2 mg/kg	Lumbosacral	Laparo-ovariectomy.	No beneficial effect over epidural injection of lignocaine alone. Duration of analgesia was 133 ± 19.5 min.	Pain was assessed in study.	10	[52]
Buprenorphine	Partial μ and κ agonist, δ antagonist	10 µg/kg	Epidural	No procedure.	Not recorded.	No.	14	[53]
		0.01 mg/kg q 8 h for 48 h beginning 1 h before anaesthesia induction.	Intravenous bolus	Instrumentation of the foetus.	Acceptable postoperative analgesia.	Physiologic variables and behavioural were recorded.	6	[54]
Buprenorphine (Slow release/long acting 72 h)	Partial μ and κ agonist, δ antagonist	0.27 mg/kg	Intramuscular	A thermal portable device was used to assess SRB-induced antinociception.	Well-tolerated analgesic. Plasma concentrations increased; the thermal withdrawal time declined.	SRB-induced antinociception.	4	[55]
		4 mg/sheep pre- and postoperatively.	Subcutaneous	Third-degree flame skin burn and smoke inhalation.	Not recorded.	None.	11	[56]
Buprenorphine and Ketamine	Partial μ and κ agonist, δ antagonist + NMDA receptor antagonist	10 µg/kg + 1 mg/kg 30 min later. Then Ketamine at 5 mg/kg/h.	Intravenous	Experimental intervertebral disk nucleotomy.	Prevented increases in HR and MAP during surgery.	Cardiovascular response to noxious stimulation.	18	[57]
Morphine	μ agonist	0.1 mg/kg post operatively	Epidural	Caesarean section.	Analgesia up to 6 h.	Adaptation of the UNESP-Botucatu One-Dimensional Scale for Post-Operative Pain Evaluation in Bovine was used.	3	[51]
		0.1 mg kg	Thoracic epidural	No procedure.	Average duration of analgesia was 45 min.	Pain was assessed in study.	6	[58]
		0.5 mg/kg	Intramuscular (preoperative)	Stifle surgery.	Effective analgesia.	Behavioural and physiological parameters were recorded.	30	[48]
		0.1 mg/kg every 4 h post-op.	Not recorded	Laparotomy and hysterectomy.	Not recorded.	Pain scores were recorded.	6	[59]
		0.2 mg/kg post-op	Intravenous	Elective laparoscopy.	Not recorded.	The UNESP-Botucatu composite scale was used to assess acute postoperative abdominal pain.	48	[37]
Methadone	μ agonist + NMDA antagonist	0.3 mg/kg	Intravenous	Experimental intervertebral disk nucleotomy.	Prevented increases in HR and MAP during surgery.	Cardiovascular response to noxious stimulation.	18	[57]
		0.3 mg/kg	Lumbosacral epidural	No procedure.	Duration of analgesia was 220 min.	Pain scored by deep application of muscle pricks.	6	[60]
Methadone and Bupivacaine	μ agonist and NMDA antagonist+ local anaesthetic	0.15 mg/kg + 0.25 mg/kg	Lumbosacral epidural	No procedure.	Duration of analgesia was 180 min.	Pain scored by application of deep muscle pricks.	6	[60]
Fentanyl	μ agonist κ agonist	2 µg/kg followed by 10 µg/kg/h	Intravenous	Experimental intervertebral disk nucleotomy.	Prevented increases in HR and MAP during surgery.	Cardiovascular response to noxious stimulation.	18	[57]
		2 µg/kg/h	Transdermal patch foreleg and thorax	No procedure.	Provided sufficient analgesia if applied 3–6 h before painful event. Foreleg patch provided faster and longer lasting analgesia.	Measured blood levels to assess if fentanyl plasma concentrations had reached the minimum analgesia level for opioid-naïve humans of 0.6–1.5 ng/mL. Physiological parameters and behaviour were observed.	12	[61]
		2 patches: 100 ug/kg/h and 50 ug/kg/h placed 1 day pre-operatively	Transdermal patch on both forelimbs	Stifle surgery.	Effective analgesia.	Behavioural and physiological parameters were recorded.	30	[48]
		2 µg/kg/h	Transdermal patch on foreleg	Orthopaedic surgery.	Minimum dose rate of 2 µg/kg was required for analgesia.	Measured blood levels to assess fentanyl plasma concentrations. Physiological parameters and behaviour were observed.	8	[62]
		2.0 μg/kg loading dose followed by 2.5 μg/kg/hr infusion	Intravenous	Abdominal surgery.	Not recorded.	No.	10	[13]
		2 μg/kg/hr	Transdermal patch	Abdominal surgery.	Not recorded.	No.	10	[13]
		1.4 ± 0.2 μg kg/hour	Transdermal patch	Laparotomy and hysterectomy.	Not recorded.	No.	10	[14]
		75 μg/hour patch	Transdermal patch	Laparotomy and hysterectomy.	No analgesia noted.	Thermal and mechanical thresholds were measured.	8	[63]
		2 μg of /kg/h	Transdermal patch	Surgery for instrumentation of the foetus.	Acceptable postoperative analgesia.	Physiologic variables and behavioural changes indicative of pain were assessed.	6	[54]
Remifentanil	μ agonist	0.33 µg/kg/min for 1 h	Intravenous continuous infusion	Caesarean section.	Not recorded.	No.	7	[15]
Oxycodone	μ agonist	0.1 mg/kg infusion then 0.05 mg/kg/h for five days.	Epidural	Laparotomy.	Not recorded.	No.	10	[16]
		Initial 0.4 mg/kg bolus followed by 0.2 mg/kg boluses BID for five days.	Epidural	Laparotomy.	Not recorded.	No.	10	[16]

**Table 3:** Local anaesthetics used for analgesia in sheep.

Drug	Action	Dose	Route	Indication	Summary of Effect	Pain Assessment Method	Sheep (n)	Reference
Lignocaine (2%) *	Local anaesthetic	2 mg/kg	Intrafunicular	Orchiectomy.	No analgesia.	Pain was assessed in study.	6	[41]
		30 mg/site	Numnuts^®^ device injection at ring site	Castration and tail docking with rubber rings.	Provided analgesia during the acute pain response.	Time to mother up, acute pain-related behaviours and postures were recorded.	50	[64]
		1.5 mL/site	Numnuts^®^ device injection at ring site	Tail docking with rubber rings.	Abolished abnormal behaviours and signs of pain however some evidence of residual discomfort remained.	Pain-related behaviours were recorded.	10	[65]
		1.5 mL/site	Numnuts^®^ device injection at ring site	Ring castration and tail docking.	Early onset but short-lived analgesia.	Active pain avoidance behaviours were recorded.	56	[66]
		8 mL/sheep	4-point regional nerve block distal to the fetlock	Single distal limb lameness.	Resulted in anaesthesia of the distal limb.	A pressure algometer was used to quantify analgesia.	18	[67]
		9 mL total. 3 mL per paravertebral nerve.	Paravertebral	Nociceptive stimuli.	Durations of analgesia was 65 ± 18 min.	Nociceptive effects were recorded.	6	[68]
		1.2 mg/kg	Lumbosacral epidural	No procedure.	Antinociceptive effects were observed up to 60 min.	Anti-nociceptive effects were recorded.	6	[69]
		2 mL/sheep	Subcutaneous–Metacarpi block	Nociceptive stimuli.	Analgesia of the Metacarpi was limited to 60 min.	Nociceptive threshold was measured.	4	[70]
		2.86 mg/kg	Lumbosacral epidural	Needle pricks.	Duration of analgesia 54.43 ± 3.28 min	Analgesia tested by recording response to sharp needle pricks.	7	[50]
		2 mL/sheep	Intra-testicular injection	Castration.	Not recorded.	Electroencephalography was used.	8	[71]
		1 mL/sheep	Subcutaneous into scrotal neck, spermatic cords, and tail prior to procedure	Ring castration and hot iron docking.	Reduced acute pain to some degree.	Behavioural indicators of pain were recorded.	15	[34]
		2 mL/sheep	Proximal paravertebral block	Caesarean section.	Not recorded.	No.	5	[51]
		2.5 mL + 5 mL/sheep	Subcutaneous (spermatic cords and scrotal neck)	Burdizzo castration.	Some analgesia within the first 2 h.	Behaviour, inflammation, ANS, HPA, and oxidative stress were recorded.	24	[45]
		Not recorded	Subcutaneous and Intramuscular inverted L block	Surgical placement of rumen fistula.	Not recorded.	Analgesia tested by recording response to sharp needle pricks.	13	[32]
		5 mg/kg	Brachial plexus block	Pin pricks and skin pinching with haemostats.	Produced forelimb analgesia within 11.3 min. Mean duration of analgesia was 100 min.	Responses to aversive pin pricks and skin pinches were recorded.	9	[72]
		5 mg/kg	Brachial plexus block	Pin pricks and skin pinching with haemostats.	Provided analgesia for 100 ± 38 min.	Responses to aversive pin pricks and skin pinches were recorded.	7	[73]
		5 mg/kg + 0.05 mg kg	Brachial plexus block	Pin pricks and skin pinching with haemostats.	Produced forelimb analgesia within 7 min. Mean duration of analgesia was 186.8 min.	Responses to aversive pin pricks and skin pinches were recorded.	9	[72]
Lignocaine and Xylazine	Local anaesthetic + α2 agonist	3.9 mg/kg + 0.05 mg/kg	Lumbosacral epidural	No procedure.	Provided prolonged anaesthesia that may contribute to pain relief in the immediate post-operative period.	Pain scoring system was used.	6	[74]
		5 mg/kg + 0.1 mg/kg	Brachial plexus block	Pin pricks and skin pinching with haemostats.	Mean duration of analgesia to brachial plexus was 103 ± 35 min. Produced forelimb analgesia within 11 min. Mean duration of analgesia was 133.2 min.	Responses to aversive pin pricks and skin pinches were recorded.	7	[73]
Lignocaine and Morphine	Local anaesthetic + μ agonist	5 mg/kg + 5 µg mL	Brachial plexus block	Pin pricks and skin pinching with haemostats.	Rapid onset analgesia with short duration of action. Duration of analgesia was 119.4 ± 52.5 min.	Responses to aversive pin pricks and skin pinches were recorded	9	[72]
Lignocaine and Adrenalin	Local anaesthetic + sympathomimetic	4 mg/kg	Lumbosacral epidural	Laparo-ovariectomy.	Not suitable for medium to long-term surgery.	Pain was assessed in study.	10	[52]
		9 mL total. 3 mL per paravertebral area.	Paravertebral	Nociceptive stimuli.	Durations of analgesia 95 ± 46 min.	Nociceptive stimuli response was recorded.	6	[68]
		4.2 mg/kg + 5 μg/mL	Lumbosacral epidural	Pin pricks and skin pinching with haemostats.	Provided prolonged anaesthesia that may contribute to pain relief immediately postoperatively.	Pain scoring was used.	6	[74]
Lignocaine and Tramadol	Local anaesthetic + Weak μ agonist + serotonin	5 mg kg + 1 mg/kg	Brachial plexus block	Pin pricks and skin pinching with haemostats.	Mean duration of sensory block was 79 ± 28 min.	Response to aversive pin pricks and skin pinches were recorded.	7	[73]
	reuptake inhibitor	2.46 mg/kg + 1 mg/kg	Lumbosacral epidural	Pin pricks with needles.	Rapid onset of perineal and cutaneous analgesia 5.58 ± 0.40 min and prolonged duration 100.7 ± 4.80 min.	Needle prick response was recorded.	7	[50]
		0.5 mg/kg	Femoral nerve or the sciatic nerve block.	Surgery on the femorotibial joint.	No clear benefit of nerve block.	Physiological and behavioural measures were recorded.	15	[48]
		1.5 mL/site	Numnuts^®^ device injection into ring site	Rubber ring castration and tail docking.	More sustained analgesia than only lidocaine.	Active pain avoidance behaviours were recorded.	32	[66]
Bupivacaine (0.75%)	Local anaesthetic	1.2 ± 0.1 mg/kg.	Lumbosacral epidural	Pin pricks and skin pinching with haemostats.	Prolonged anaesthesia that might contribute to pain relief in the postoperative period.	Pain scoring was used.	6	[74]
Bupivacaine (0.5%)	Local anaesthetic	9 mL total. 3 mL per paravertebral nerve.	Paravertebral	Nociceptive stimuli.	Produces a longer duration of analgesia than lidocaine with or without epinephrine.	Nociceptive response to stimuli.	6	[68]
		1.25 mg/kg	Brachial plexus block	Pin pricks and skin pinching with haemostats.	Mean duration of sensory block was 335 ± 134 min.	Responses to aversive pin pricks and skin pinches were recorded.	7	[73]
		2 mL/site	Subcutaneous–Metacarpal block	Nociceptive stimuli.	Duration of anaesthesia 110.0 ± 47.26 min. Lasted for 120 min, and the best analgesia was between 60 and 120 min.	Nociceptive threshold was measured.	4	[70]
		0.5 mg/kg	Lumbosacral epidural	Deep muscle needle pricks.	Duration of analgesia was 240 min.	Response was scored after deep muscle pricks.	6	[60]
		0.5 mg/kg	Thoracic epidural	No procedure.	Average duration of analgesia was 60 min.	Pain was assessed in study.	6	[58]
Bupivacaine (0.25%)	Local anaesthetic	0.5 mg/kg	Lumbosacral epidural	Painful stimulus.	Duration of analgesia was 240 min.	Response to painful stimulus was recorded.	6	[11]
Bupivacaine and Morphine	Local anaesthetic + μ agonist	0.25 mg/kg + 0.05 mg kg	Thoracic epidural	No procedure.	Average duration of analgesia was 140 min.	Pain was assessed in study.	6	[58]
Bupivacaine and Lignocaine	Local anaesthetics	1 mL + 11 mL/ sheep	Subcutaneous metacarpal ring block	Nociceptive stimuli.	Anaesthesia lasted twice as long than with lignocaine alone. Onset of analgesia was slower than bupivacaine alone.	Nociceptive threshold was measured.	4	[70]
Bupivacaine and Methadone	Local anaesthetic + μ agonist and NMDA antagonist	0.25 mg/kg + 0.3 mg/kg	Lumbosacral epidural		Duration of analgesia was 240 min.	Response to a painful stimulus was recorded.	6	[11]
Bupivacaine and Fentanyl	Local anaesthetic + μ agonist and κ agonist	0.25 mg/kg + 0.002 mg/kg	Lumbosacral epidural	Painful stimulus..	Duration of analgesia was 180 min.	Response to a painful stimulus was recorded.	6	[11]
Levobupivacaine	Local anaesthetic	0.05 mg/kg	Lumbosacral epidural	Deep muscle needle pricks.	30 ± 5 min of local anaesthesia.	Response to deep muscle pricks were recorded.	6	[75]
		0.15 mg/kg	Lumbosacral epidural	Deep muscle needle pricks.	145 ± 27 min of local anaesthesia.	Response to deep muscle pricks were recorded.	6	[75]
		0.25 mg/kg	Lumbosacral epidural	Deep muscle needle pricks.	290 ± 18 min of local anaesthesia.	Response to deep muscle prick was used as a painful stimulus.	6	[75]
Procaine (5%) and adrenalin (0.002%)	Local anaesthetic + sympathomimetic	0.3 mL/ lamb (2- to 3-day-old lambs) at time of procedure	Subcutaneous injection into Spermatic cords	Castration with rubber rings.	Produced acute analgesia for visceral pain.	Active behavioural responses and postures of the lambs were recorded.	8	[76]
		1.5 mL/site (75 mg per site)	Numnuts^®^ device injection into ring site	Castration and tail docking using rubber rings.	More sustained and quicker onset of analgesia than lidocaine.	Active pain avoidance behaviours were recorded.	17	[66]
Ropivacaine (0.5%)	Local anaesthetic	10 mL/sheep	Block of the femoral and sciatic nerves under ultrasound guidance	Tibial osteotomy.	Analgesia for an average of 6 h.	Grimace scale, pain scoring, heart rate, respiratory rate, and mean blood pressure were recorded.	12	[9]
		10 mL/sheep	Epidural	Tibial osteotomy.	Analgesia for an average of 8 h.	Grimace scale, pain scoring, heart rate, respiratory rate, and mean blood pressure were recorded.	13	[9]
Tri-Solfen^®^ * (Lignocaine, bupivacaine, adrenalin and cetrimide)	Local anaesthetic + sympathomimetic + antiseptic	Single spray of 1.5 mL/sheep applied to lesions	Topical	Treatment of Orf virus lesions.	Not recorded.	No.	11	[77]
		0.5 mL/kg	Topical spray onto wound	Mulesing + hot-iron tail docking.	Significant analgesia for at least 24 h after mulesing.	Body weight, behavioural responses, assessment of skin and wound sensitivity, and time to mother up and to feed were measured.	20	[78]
		Lambs 5–10 kg: 6 mL 11–15 kg: 8 mL 16–20 kg: 10 mL >20 kg: 12 mL	Topical spray onto wounds	Surgical mulesing and hot-knife tail docking.	Provided analgesia. Pain was not eliminated.	Lamb behaviour was observed.	20	[23]
		Lambs 5–10 kg 6 mL; 11–15 kg 8 mL; 16–20 kg 10 mL; >20 kg 12 mL	Spray onto wounds	Surgical mulesing and hot-knife tail docking.	Provided rapid-onset analgesia.	Pain avoidance behaviour, cortisol concentrations and postural behaviour were recorded.	24	[23]

* = registered for use in Australia.

**Table 4:** α_2_ Adrenoreceptor agonists used for analgesia in sheep.

Drug	Action	Dose	Route	Indication	Summary of Effect	Pain Assessment Method	Sheep (n)	Reference
Clonidine, Lignocaine (2%) and Buprenorphine	α_2_ agonist + local anaesthetic + Partial μ and κ agonist and δ antagonist	2 µg/kg + 2 mg/kg + 300 µg	Intrathecal	Spinal anaesthesia for orthopaedic surgery.	Addition of clonidine produces a faster onset and a long-lasting analgesia compared to lidocaine and buprenorphine combination.	Presence of reflexes were assessed, and ataxia was scored.	20	[79]
Xylazine *	α_2_ agonist	0.4 mg/kg	Intramuscular	Skin and muscle needle pricks.	Xylazine has a mild analgesic effect on sheep during deep sedation.	Skin and muscle pricks were used as a painful stimulus.	5	[80]
		0.2 mg/kg	Intravenous	No procedure.	Produced skin analgesia and medium to deep degree of sedation.	Analgesic effects were recorded.	8	[81]
Medetomidine	α_2_ agonist	15 μg/kg	Intravenous	No procedure.	Not recorded.	No.	4	[82]
		15 μg/kg	Oral	No procedure.	Not recorded.	No.	4	[82]
		6 μg/kg	Intravenous	No procedure.	No analgesia was achieved after administration. Produced light to medium sedation.	Pain scoring was performed.	8	[81]
		3 µg/kg/h	Intraperitoneal (continuous infusion) postoperatively	Laparotomy and hysterectomy.	Provided analgesia for 24 h after surgery.	Pain was assessed.	6	[59]
		3 μg kg/hour	Intraperitoneal via osmotic pump	Laparotomy and hysterotomy.	May have a role in providing post-operative analgesia.	Thermal and mechanical thresholds were recorded.	8	[63]
Dexmedetomidine	α_2_ agonist	2.5 μg/kg	Lumbosacral epidural	Nociceptive stimuli.	Inferior antinociceptive effects compared to dexmedetomidine and lignocaine combination.	Anti-nociceptive effects were measured.	6	[69]
		1 μg/kg/h for 3 h	Intravenous	No procedure.	No recorded.	No.	1	[83]
Dexmedetomidine and Lignocaine	α_2_ agonist + local anaesthetic	2.5 μg/kg + 1.2 mg/kg	Lumbosacral epidural	Nociceptive stimuli.	Prolonged analgesia	Anti-nociceptive effects were measured.	6	[69]
Detomidine	α_2_ agonist	40 μg/Kg	Intravenous	No procedure	No analgesia was achieved after administration. Produced light to medium sedation.	Analgesic effects were recorded.	8	[81]

* = registered for use in Australia.

**Table 5:** NMDA Receptor Agonists and Other Analgesics in sheep.

Drug	Action	Dose	Route	Indication	Summary of Effect	Pain Assessment Method	Sheep (n)	Reference
Metamizole	Non opioid analgesic	1000 mg/sheep SID	Intravenous	Post-op analgesia.	Effect not recorded.	No.	10	[7]
Ketamine *	NMDA receptor antagonist	2.5 mg/kg	Lumbosacral epidural	Deep muscle needle pricks.	41 ± 7 min of analgesia.	Deep muscle pricks were used as a painful stimulus.	6	[84]
Racemic ketamine and Lignocaine (2%)	NMDA receptor antagonist + local anaesthetic	3.0 mg kg + 1.5 mg kg	Subarachnoid	Bilateral orchiectomy.	Produced surgical analgesia and recumbency.	Response to scrotal skin pricks recorded.	10	[44]
Ketamine and Magnesium Sulphate	NMDA receptor antagonists	2.5 mg/kg + 100 mg	Lumbosacral epidural	Deep muscle needle pricks.	115 ± 17 min of analgesia.	Response to deep muscle pricks recorded.	6	[84]
Magnesium sulphate	NMDA receptor antagonist	100 mg/sheep	Lumbosacral epidural	Deep muscle needle pricks.	29 ± 5 min of analgesia.	Response to deep muscle pricks recorded.	6	[84]
Proglumide	Inhibitor of Cholecystokinin	25 or 50 µg/kg	Intracerebroventricular	Mechanically induced duodenal distension.	Effective analgesic agent for duodenal pain.	Sheep behaviour, plasma catecholamines (CA), cortisol concentration, and clinical symptoms of visceral pain.	6	[85]
Diltiazem	Voltage-Dependent Calcium Channel Inhibitor	25 or 50 µg/kg	Intracerebroventricular	Mechanically induced duodenal distension.	Prevented nocifensive signs of behaviour and clinical symptoms, as well as increased plasma cortisol and catecholamine concentration in periphery and perhaps in CNS structures.	Sheep behaviour, plasma catecholamines (CA), cortisol concentration, and clinical symptoms of visceral pain.	6	[85]
Nifedipine	Voltage-Dependent Calcium Channel Inhibitor	25 or 50 µg/kg	Intracerebroventricular	Mechanically induced duodenal distension.	Provided peripheral analgesia and possibly CNS analgesia.	Sheep behaviour, plasma catecholamines (CA), cortisol concentration, and clinical symptoms of visceral pain.	6	[85]
Verapamil	Voltage-Dependent Calcium Channel Inhibitor	25 or 50 µg/kg	Intracerebroventricular	Mechanically induced duodenal distension.	Provided peripheral analgesia and possibly CNS analgesia.	Sheep behaviour, plasma catecholamines (CA), cortisol concentration, and clinical symptoms of visceral pain.	6	[85]
L-AP_3_	Inhibitor of Metabotropic Glutaminergic Receptors (mGluR_1)_	0.2, 0.4, and/or 0.8 mg total/sheep	Intracerebroventricular	Mechanically induced duodenal distension.	Worked as an analgesic and an antistress agent.	Sheep behaviour, plasma catecholamines (CA), cortisol concentration, and clinical symptoms of visceral pain.	6	[85]
DL-AP_3_	Inhibitor of Metabotropic Glutaminergic Receptors (mGluR_1)_	2, 4, and/or 8 mg total/sheep	Intracerebroventricular	Mechanically induced duodenal distension.	Worked as an analgesic and an antistress agent.	Sheep behaviour, plasma catecholamines (CA), cortisol concentration, and clinical symptoms of visceral pain.	6	[85]
Salicylic Acid	Monohydroxybenzoic acid, nonselective COX inhibitor	10, 50, 100, and 200 mg/kg	Intravenous	No procedure.	Not recorded.	No.	6	[86]
		100 and 200 mg/kg	Oral	No procedure.	Not recorded.	No.	6	[86]
Paracetamol	Non NSAID analgesic and anti-pyretic	10 mg/kg	Intravenous	Post-surgical analgesia.	Not recorded.	Undisclosed.	7	[87]
		15 mg/kg orally BID for 6 doses	Oral	Post-surgical analgesia.	Not recorded.	Undisclosed.	7	[87]
Amitriptyline	Tricyclic antidepressant	5 mg/sheep	Intravenous	No procedure.	Not recorded.	Undisclosed.	6	[88]
		10 mg/sheep	Intrathecal	No procedure.	Not recorded.	Undisclosed.	6	[88]
		50 mg/sheep	Epidural	No procedure.	Not recorded.	Undisclosed.	6	[88]

* = registered for use in Australia.

## Data Availability

The raw data supporting the conclusions of this article will be made available by the authors on request.
